# ConA-Like Lectins: High Similarity Proteins as Models to Study Structure/Biological Activities Relationships

**DOI:** 10.3390/ijms20010030

**Published:** 2018-12-21

**Authors:** Benildo S. Cavada, Vanir R. Pinto-Junior, Vinicius J. S. Osterne, Kyria S. Nascimento

**Affiliations:** BioMol-Lab, Department of Biochemistry and Molecular Biology, Federal University of Ceara, Fortaleza-CE 60440-970, Brazil; juniorreis4@hotmail.com (V.R.P.-J.); vinnyosterne@gmail.com (V.J.S.O.); kyriasantiago@gmail.com (K.S.N.)

**Keywords:** lectins, ConA-like, diocleinae subtribe, structure, biological activities

## Abstract

Lectins are a widely studied group of proteins capable of specific and reversible binding to carbohydrates. Undoubtedly, the best characterized are those extracted from plants of the Leguminosae family. Inside this group of proteins, those from the Diocleinae subtribe have attracted attention, in particular Concanavalin A (ConA), the best-studied lectin of the group. Diocleinae lectins, also called ConA-like lectins, present a high similarity of sequence and three-dimensional structure and are known to present inflammatory, vasoactive, antibiotic, immunomodulatory and antitumor activities, among others. This high similarity of lectins inside the ConA-like group makes it possible to use them to study structure/biological activity relationships by the variability of both carbohydrate specificity and biological activities results. It is in this context the following review aims to summarize the most recent data on the biochemical and structural properties, as well as biological activities, of ConA-like lectins and the use of these lectins as models to study structure/biological activity relationships.

## 1. Introduction

Widely distributed in nature, lectins are a heterogeneous group of proteins that can be found from the simplest organisms, such as viruses, to animals and plants [1,2]. During the evolutionary process, these proteins retained a common feature: the ability to recognize and bind reversibly to specific carbohydrates without structurally altering the carbohydrate [3,4]. According to Peumans and Van Damme (1995) [3], lectins can be defined as a class of proteins of nonimmune origin capable of reversibly binding to specific mono- or oligosaccharides through a noncatalytic domain.

Legume lectins represent the most well-studied group among plant lectins. Most studies have reported on lectins from the subfamily Papilionoideae, mainly the tribe Phaseoleae [5]. Within this tribe, we can highlight the subtribe Diocleinae that presents 13 taxa at genus level: *Canavalia*, *Dioclea*, *Cymbosema*, *Cleobulia*, *Macropsychanthus*, *Neorudolphia*, *Lackeya*, *Luzonia*, *Camptosema*, *Cratylia*, *Collaea*, *Rhodopis* and *Galactia* [6,7].

Lectins of this subtribe have high structural similarity, some presenting differences that separate one from another by only three residues, but, despite such small differences, these lectins still present different effects in biological activities, a fact first reported in the work of Cavada et al. (2001) [8]. Within this group of lectins is found Concanavalin A (ConA), a mannose/glucose-specific lectin. ConA is purified from *Canavalia ensiformis* seeds and is the most widely studied lectin [9]. Diocleinae lectins closely related to ConA are commonly called ConA-like lectins.

These proteins are known to possess inflammatory, nociceptive, vasoactive, antiproliferative, antimicrobial, immunomodulatory, and antidepressant activities, among others [8,10]. Most of these biological activities are directly related to the ability of these proteins to interact with carbohydrates via the carbohydrate recognition domain (CRD) [11]. This interaction can be influenced by CRD conformation, which is defined by the amino acid composition, as well as the oligomerization state of these molecules, which may be dependent on the pH of the medium [12]. High similarity, together with variability of biological effects, makes this group of lectins useful for the study of structure/biological activity relationships since the impact of even small structural changes can be correlated with the type and/or intensity of the biological response [8].

It is in this context that the following review summarizes the most recent studies of ConA-like lectins from the perspective of structure/biological activity relationships.

## 2. Carbohydrate-Specificity Overview

Several works have addressed the carbohydrate specificity of ConA-like lectins by employing several methods [13,14,15]. The specificity of ConA-like lectins for monosaccharides is well defined with all lectins presenting preferential binding to mannose, glucose and derivatives without binding to galactose, fucose or sialic acid. It is reported that the C-2 hydroxyl group of mannose is not essential for binding, while C-3, C-4 and C-6 are. For Methyl α-D-mannopyranoside, the most specific monosaccharide, the free energy of binding for different ConA-like lectins ranges from −4.4 to −5.62 kcal/mol. For disaccharides, ConA-like lectins have a preference for diglucosides and dimannosides, especially mannosyl-α1,2-mannose and mannosyl-α1,6-mannose. All tested ConA-like lectins recognized the core trimannoside of N-linked glycans. These lectins could recognize 3-, 4- and 6-hydroxyl groups of α(1-6)-Man, 3- and 4-hydroxyl groups on α(1-3)-Man, and 3- and 4- hydroxyl groups of the central mannose residue from trimannosides [14]. Insights into the fine specificity of ConA for glycans were obtained by Maupin and colleagues (2012) [15] who employed glycan array data and outlier-motif analysis with several *N*-glycans. The authors observed very high scores for the binding with glycans containing Manα on terminal or subterminal position, followed by glycans with Glcα on terminal position. They also observed that the presence of phosphate on the mannose significantly reduced the binding.

In the work of Ramos et al. (2002) [16], the interaction of several lectins with glycoproteins was investigated by surface plasmon resonance. Results showed that all tested ConA-like lectins interacted with the tested glycoproteins with affinity varying from lectin to lectin. Tested glycoproteins included *Phaseolus vulgaris* erythroagglutinin (PHA-E), Soybean agglutinin (SBA), arcelin-1, hen ovalbumin, orosomucoid or acid α-glycoprotein, ovomucoid, bovine lactotransferrin and human serotransferrin. The differences in binding are associated with small differences among these proteins.

## 3. Overview of ConA-Like Lectins Purification

Many ConA-like lectins have already been purified, mostly from seeds, indicating, in most cases, a largely standardized protocol. Generally, purification of a Diocleinae lectin involves the following steps. First, seeds are peeled and ground until obtaining a fine powder. Second, the soluble proteins are extracted, either in buffer or a saline solution, with or without CaCl_2_ and MnCl_2_. The crude extract can then be submitted to a fractionation step with ammonium sulfate precipitation or directly applied on an equilibrated affinity chromatography with matrix of immobilized mannose or dextran. Sephadex G-50 is the most common. The unretained peak is eluted with the equilibration solution, and the bound protein is eluted by competition with mannose or glucose on the equilibration solution/buffer, or by reversible denaturation with a mild acidic buffer, such as glycine-HCl, pH 2.6 [17,18,19,20]. This protocol usually results in a high degree of purity, but further purification steps by gel filtration and/or ion exchange chromatography can be employed.

Instead of affinity chromatography, the aqueous two-phase system (ATPS) can be used. This is a scalable and low-cost system adequate for large-scale purification systems [21]. It has been employed for ConA [22,23], *Canavalia brasiliensis* lectin (ConBr) [24,25] and *C. grandiflora* lectin (ConGF) [26] with good results. The most successful systems for the purification of ConA-like lectins include PEG–dextran, PEG–citrate and PEG–phosphate [21]. Other methods, such as membranes, have also been employed for ConA purification [27].

## 4. Biological Activities of ConA-Like Lectins

### 4.1. Inflammatory and Nociceptive

Many Diocleinae lectins have demonstrated anti- or proinflammatory effects in murine models, depending on the administration route used in the assay. When the administration route was local, these lectins elicited paw edema and neutrophil migration. On the other hand, when administered systemically, they exhibited anti-inflammatory activity with increased cellular permeability, stimulating neutrophil migration [28,29]. In a study by Assreuy et al. (1997) [28], all tested lectins had anti-inflammatory effect, except for ConBr. This result was confirmed more recently by Pinto et al. (2013) [30] and has been attributed to small structural differences between these lectins which may involve interaction with glycans.

It has been proposed that the triggering mechanisms of ConA’s inflammatory effect include the binding to glycans present on the surface of endothelial cells and capturing of neutrophils to begin the migration process [28,31,32,33]. Anti-inflammatory effect could be explained by the competition of exogenous lectins with selectins (L-, P- and E-selectin) for glycosylated sites on leukocyte and/or endothelial cell membranes [33,34].

Edematogenic activity has been studied in ConA [31], ConGF [35], ConBr [29], *Canavalia gladiata* lectin (CGL) [29], *C. maritima* lectin (ConM) [29], *C. virosa* lectin (ConV) [36], *C. oxyphylla* lectin (CoxyL) [19], *C. villosa* lectin (CvilL) [20], *Dioclea rostrata* lectin (DRL) [37], *D. wilsonii* lectin (DWL) [38,39], *D. virgata* lectin (DvirL) [40], *D. lasiophylla* lectin (DlyL) [41], *D. reflexa* lectin (DrfL) [41] and *Cymbosema roseum* lectin (CRL1) [42] (Table 1). Anti-inflammatory effect has been studied in ConGF [43], DvirL [33], CRL1 [42], *D. guianensis* lectin (DguiL), *D. violacea* lectin (DVL) and *Cratylia floribunda* lectin (CFL) [28].

Many inflammatory processes promoted by these lectins are accompanied by hypernociception. For example, when injected subcutaneously intraplantar in rats, DlyL and CvilL induced paw edema and increased the sensitivity of paws, as evaluated by the application of von Frey filaments [20,44]. However, when administered intravenously or orally, they demonstrate antinociceptive effects. For example, ConGF inhibited inflammatory hypernociception in the von Frey test [43], whereas DvirL inhibited paw licking in the formalin test [40], and *Canavalia boliviana* lectin (CboL) has exhibited central and peripheral nociception in several models [37]. Holanda et al. (2009) [45] studied ConM, ConGF, DguiL, DVL and CFL in tests using a model of acetic acid-induced abdominal constriction and showed antinociceptive responses when given orally. Additionally, ConBr exhibited antinociceptive properties mediated by the opioid system after oral administration [46].

Although they have high structural similarity, these lectins demonstrate different potency, efficacy and mechanisms in their biological activity, as demonstrated by comparing the studies noted above related to the results of inflammation and nociception. For instance, Pinto et al. (2013) [30] evaluated the antinociceptive effect of CGL, ConM and ConBr in several nociception models. The effects occurred predominantly via peripheral nociception and showed different results according to the potency of the elicited effect.

In most of these studies, the use of lectins preincubated with their specific carbohydrates partially inhibited the effect they promoted, demonstrating the participation of the CRD in their activities. Indomethacin and L-NAME, inhibitors of cyclooxygenase and nitric oxide synthase (NOS), respectively, two enzymes involved in nitric oxide metabolism, could also inhibit the effects of these lectins, demonstrating the participation of nitric oxide (NO).

### 4.2. Vasoactive

Diocleinae lectins have vasorelaxant potential, as shown on in vivo and in vitro assays. One of the first studies to explore this activity was performed by Kleha et al. (1991) [47] who demonstrated that ConA could relax rabbit pre-contracted aorta with phenylephrine, having a more potent effect when the endothelium was preserved. Years later, this property was studied for other lectins of this subtribe in a murine model. In 2005, Gadelha and colleagues (2005) [48] evaluated the ability of ConM to relax rat aortic rings with dose-dependent effect and demonstrated the need for the presence of endothelium for its activity. In addition, ConM demonstrated vasorelaxant potential exceeding that of ConA. Similar results were observed in other works involving other Diocleinae lectins, such as CGL [29,49], ConBr [29,49], ConV [17], CRL1 [50], *D. lasiocarpa* (DLL) [51], DrfL [52], *D. sclerocarpa* (DSL) [53], DVL [54] and DRL [54], with different activity intensities (Table 2).

Enzyme inhibitors of NO metabolism have either partially or totally blocked the vasorelaxant effect of these lectins, demonstrating the effect of endothelial NO on the activity of these proteins. NO is a relaxing factor derived from the endothelium. Endothelial NOS (eNOS) is activated in response to shear stress and numerous agonists by means of cellular events, such as interaction with substrate and cofactors, increased intracellular calcium, as well as protein phosphorylation and transport between distinct subcellular domains [55,56].

How these lectins activate eNOS is still unclear, but it is well known that such activity can be reversed by pre-incubation of these lectins with their specific carbohydrates, which has allowed deducing that eNOS activation likely derives from the interaction between lectins and a glycosylated receptor present on the surface of endothelial cells. The form and/or intensity of interaction of each lectin with that receptor may account for the difference in intensity.

### 4.3. Antiproliferative

Studies have reported on the application of plant lectins as anticancer agents owing to their potent inhibitory effects, cytotoxicity and the ability to induce apoptosis and autophagy in several tumor cell lines [57,58]. These lectins can inhibit tumorigenesis by binding to glycosylated proteins on the membrane of cancer cells. It is well known that glycoconjugates play important roles in many biological processes, including cancer, with malignant cells usually presenting altered glycosylation patterns [11]. These changes in glycans allow preferential binding of lectin on cancer cells to induce the above effects.

Recent studies demonstrated that Diocleinae lectins, such as ConV, *C. bonariensis* lectin (CaBo), DVL and DLL, could induce cell death in glioma cells, especially the C6 rat glioma strain. All of these lectins induced a significant reduction in cell viability ranging from 30% to 60% with 100 μg/mL concentration and 48 h exposure. Some of these lectins affected mitochondrial membrane potential or cell migration, and all of them induced morphological changes on cells from a polygonal form to a spherical one, followed by reduction of cell adhesion. Staining methods indicated cell death by autophagic, necrotic and apoptotic processes induced by mitochondrial pathways and modulation of metalloproteinases [36,59,60,61]. Table 3 summarizes the antiglioma activities of the lectins cited above.

Liu and colleagues (2009) [62] tested the antiproliferative effect of ConA against human melanoma A375 cells. Results show that ConA presented CRD-dependent cytotoxic effect against the tested cells in a dose- and time-dependent manner with IC_50_ of 25 μg/mL in 24 h. Morphological changes, including membrane blebbing and nuclear condensation, were observed. The number of apoptotic cells was also upregulated with a lower count of necrotic cells, suggesting that cell death was caused by apoptosis. The authors verified that the lectin treatment induced mitochondrial membrane potential collapse and cytochrome c release that caused caspase activation, leading to the conclusion that ConA-induced cell death occurred via a mitochondrial apoptotic pathway.

Gondim et al. (2017) [63] also investigated the antiproliferative potential of the lectins from DLL, ConM, DSL and ConBr by evaluating the effects of apoptosis and cell cycle on ovarian, lung, prostate, and breast cancer cell lines. In general, all tested lectins presented significant effects against ovarian, lung and prostate cancer cell lines with DLL exhibiting the lowest IC_50_ of 52 ± 2 nM against A2780 ovarian cancer cells. The lectin appeared to cause morphological changes in the cancer cells with enlargement of the nucleus and cell spreading. A closer examination of the triggering mechanisms showed that DLL induced G2/M cell cycle arrest with a large increase in Caspase-9 activation, indicating programmed cell death by apoptosis.

### 4.4. Antibacterial and Antifungal

Some ConA-like lectins act as antibacterial and/or antifungal agents. Cavalcante et al. (2011) [64] tested the effect of several *Canavalia* lectins against oral Streptococci. For *Streptococcus mutans*, CboL, ConBr and ConM presented antibacterial effect, while CGL and ConA stimulated bacterial growth. For *S. oralis*, CboL, ConBr, CGL and ConM stimulated bacterial growth, while ConA had no effect. CboL, ConA and ConM inhibited biofilm formation of *S. mutans*, while none of the other tested lectins affected *S. oralis* biofilm formation. The authors suggested that these differences resulted from variable distances between amino acid side chains on CRD, which, in turn, changed the aperture and deepness of the site.

Gomes et al. (2012) [65] reported on the antifungal effect of DRL, ConBr and DVL on yeasts isolated from vaginal secretions. DRL demonstrated antifungal effect against *Candida guilliermondii*, *C. shehatae*, *C. membranaefaciens* and *Kloeckera apiculata* with mean concentration until 128 μg/mL. ConBr showed antifungal activity in ten isolates, namely *C. albicans*, *C. guilliermondii*, *C. membranaefaciens*, *C. parapsilosis*, *C. shehatae*, *C. tropicalis*, *C. tropicalis*, *C. tropica*, *K. apiculata* and *Trichosporon cutaneum*, with mean concentrations ranging from 2 to 256 μg/mL. DVL inhibited the growth of *C. albicans*, *C. guilliermondii*, *C. membranaefaciens*, *C. obtusa*, *C. parapsilosis*, *C. shehatae*, *C. tropicalis*, *C. tropicalis*, *K. apiculata* and *Rhodotorula glutinis* with mean concentrations ranging from 8 to 256 μg/mL. The authors suggested that the inhibitory effect was likely caused by the inhibition of spore germination and mycelium growth by some mechanism that changed chitin synthesis and resultant deficiency in cell wall deposition. The authors concluded that these three lectins are promising for the development of therapeutic strategies against these yeasts.

### 4.5. Mitogenic

Many lectins with distinct properties and carbohydrate specificities have demonstrated mitogenic effect, such as lymphoproliferation [66,67,68]. The mitogenic elicited by these proteins is directly related to their carbohydrate-binding capacity, and the effect seems to depend on the affinity of the lectin for the glycans present on immune cell receptors.

ConA is a well-known mitogenic lectin. Beckert and colleagues (1970) [69] studied the mitogenic activity of ConA for peripheral blood lymphocytes. The authors observed a high degree of mitogenic activity on rabbit and human lymphocytes stimulated by *Canavalia ensiformis* saline extract and ConA. All cultures revealed various stages of blastoid transformation such as that seen with PHA mitogenic stimulation.

Melo et al. (2010) [70] evaluated the in vivo proliferative capacity and cytokine production of *Cratylia mollis* lectin (Cramoll), a Diocleinae lectin, for mouse splenocytes. It was verified that mice previously inoculated with Cramoll produced higher splenocyte mitogenic response with maximum proliferation induced by Cramoll in a final concentration of 10 μg/mL. This corroborated cell cycle assays that also showed higher proliferation indexes promoted by the lectin. This lectin induced more IL-6 production in comparison with IL-2, as well as high IFN-γ production, high NO production and low IL-10 release. In vitro tests indicated that Cramoll caused little damage to splenocytes in culture. Altogether, the authors suggested that Cramoll can be used as a mitogenic agent.

A third example of the mitogenic effect of a ConA-like lectin is reported in the work of Silva et al. (2011) [67] who investigated the immunostimulatory response induced by ConBr in mouse splenocyte culture. Results showed that ConBr could induce the proliferation of splenocytes in all tested concentrations and that the effect was more potent than that of ConA. ConBr induced higher IL-2, IL-6, and IFN-γ production than ConA. An elevated release of NO was induced by lectin treatment. In vitro viability assays showed that ConBr caused little damage to the cells with survival rates as high as 90%. Similar to other lectins, ConBr has an interesting mitogenic capacity.

### 4.6. Immunomodulatory

By interaction with glycans on the surface of cells of the immune system, plant lectins can act as immunomodulatory agents that can trigger cytokine production and induce optimized immune responses against some pathological conditions, such as tumors and microbial infections, enabling their application in biomedical research and therapeutics [71,72]. Inside the group of Diocleinae lectins, ConA, ConBr, Cramoll, DRL, DVL and DvirL are some examples of lectins with reported immunomodulatory activity [71,73,74,75,76].

Reis et al. (2008) [73] studied the immunomodulatory potential of several ConA-like lectins by evaluating their effect on the cytokine release of peripheral blood mononuclear cells with and without *Schistosoma mansoni* infection and then using the effect on soluble egg antigen (SEA) as a basis of comparison. ConA, ConBr, *Dioclea grandiflora* lectin (DGL), DguiL, DRL, DVL and DvirL all induced significantly higher IL-5 production on infected cells. A trend of higher interferon-γ (IFN-γ) production on uninfected cells was also elicited by the above-cited lectins. Interleukin-10 (IL-10) and tumor necrosis factor-α (TNF-α) production were not affected by the lectins.

Melo et al. (2010) [70] evaluated the immunomodulatory response induced by Cramoll on murine lymphocyte culture employing cytotoxic assays, as well as analyzing nitric oxide (NO) and cytokine production. The authors observed that Cramoll was not toxic up to a concentration of 25 µg/mL. While the lectin induced a substantial production of IFN-γ, it kept IL-10 levels low. Cramoll also showed an anti-inflammatory-like response by suppressing NO production. Another work from Silva and colleagues (2015) [77] aimed to investigate the potential immunomodulatory capacity of both native and recombinant Cramoll peritoneal exudate cells (PECs) infected, or not, with *Staphylococcus aureus*. Treatment of cells with both lectins induced a significant increase in cell viability with a concomitant increase in NO, IL-1β, IL-6, IFN-γ and TNF-α, as well as superoxide anion production by the PECs. A significant increase of *S. aureus* phagocytosis was also observed after lectin treatment, suggesting an enhanced expression of Toll-like receptor-2. The lectins also regulated cytokine production after bacterial infection, mostly TNF-α and IL-6, both important mediators of septic shock caused by *S. aureus* infection [78]. These findings support this lectin as a potent immunomodulatory protein.

### 4.7. Antidepressant and Neuroprotective

Diocleinae lectins have been applied in studies related to the central nervous system (CNS) of mammalians to further understand their function in relation to neuroplasticity [79,80,81] and to purify synaptic proteins [82,83]. In this context, some Diocleinae lectins, such as ConBr and ConA, were tested for their antidepressant effect by employing the forced swimming test on rats, an experimental procedure used for screening of compounds with antidepressant effect [84], as performed in the work of Barauna and colleagues (2006) [85]. Contrary to ConA, ConBr presented antidepressant effect with a mechanism involving serotonergic receptors (5-HT1A and 5-HT2), adrenergic receptor (or adrenoceptor) (α2-adrenoceptors), dopaminergic (D2) receptors and N-methyl-D-aspartate (NMDA) receptor [86].

ConBr induced a protective effect against seizures induced by quinolinic acid. This effect was dependent on the native structure of the lectin, and the mechanism of action appeared to be related to the block of quinolinic acid binding to NMDA receptor and/or stabilization of the receptor on an inactive conformation. ConA was also tested, but different from ConBr, it had no protective effect [87].

### 4.8. Insecticidal

The insecticidal potential of plant lectins is widely reported in the literature. The suggested mechanism, although not fully elucidated, appears to involve the binding to glycans present of the surface of the insect’s epithelial intestinal cells, leading to interference with digestive, protective and secretory processes [88,89]. Because most *N*-glycans present on the insect’s glycoproteins are of the high-mannose type, ConA-like lectins present themselves as potential insecticidal agents [90].

One example of the insecticidal potential of ConA-like lectins was published by Oliveira et al. (2015) [91]. The article investigated the insecticidal effect of DVL against *Anagasta kuehniella*, a lepidopteran pest of stored grains. DVL fed to insects caused significant weight loss in larvae, up to 55%, extension of larval stage and total development time, significant differences in food consumption by DVL-fed larvae, and reduction in enzymatic activity when compared to controls. DVL resistance to proteolysis was investigated by incubation of the lectin with midgut extract for 24 h, and DVL appeared to be highly resistant to proteolysis. Only a small amount of the lectin reached the epithelial membrane with most of it remaining confined to the endoperitrophic space and connected to the peritrophic membrane. Thus, the authors suggested that the binding to these regions can interfere with the endo-/ectoperitrophic circulation of digestive enzymes. Rahbé et al. (1995) [92] reported the effects of several lectins, including ConA, on development and survival of six aphid species. ConA was toxic to most of the tested aphids with mortality ranging from 25% to 65% at a dose of 1500 µg/mL. ConA suffered very little proteolysis in the insects’ midgut and appeared to interact with the digestive tract. Finally, Granjeiro (1996) [93] tested the insecticidal potential of ConBr against *Nilaparvata nugens* and *Aulacorthum solani*, plagues that affect rice and potato cultures, respectively. The author verified that ConBr is highly toxic to *N. nugens* nymphae with up to 64% mortality. On the other hand, *A. solani* fed with the lectin showed no effects on its growth or development.

## 5. Structure of ConA-like Lectins

### 5.1. Primary Structure

ConA primary structure was determined in 1975 by Cunningham and colleagues [94] by Edman degradation, and, since then, 22 ConA-like lectins from the Diocleinae subtribe have had their primary structures determined using Edman degradation, as well as mass spectrometry and DNA sequencing: ConV [36] (Uniprot id: C0HJY1), CGL (Uniprot id: P14894), ConM [95] (Uniprot id: P81364), ConBr [96] (Uniprot id: P55915), CboL [97] (Uniprot id: A0A023GPI8), CaBo [98] (Uniprot id: P58906), ConGF [99] (Uniprot id: A0A067XG71), DvirL (Uniprot id: P58907), CRL1 [42] (P86184), DLL [63] (PDB id: 5UUY), DlyL [44] (Uniprot id: C0HK27), DRL [100] (Uniprot id: P58908), DguiL [101] (Uniprot id: P81637), DGL [101] (Uniprot id: P08902), Cramoll [102] (Uniprot id: P83721), CFL [101] (Uniprot id: P81517), DVL (Uniprot id: I1SB09), *Camptosema pedicaellatum* lectin (CPL) [103] (Uniprot id: J9PBR3), DSL [104] (Uniprot id: B3EWJ2), DWL [38] (Uniprot id: P86624), DrfL [52] (Uniprot id: C0HK81) and *Dioclea lehmanni* lectin (DlehL) [95].

**Figure 1:**
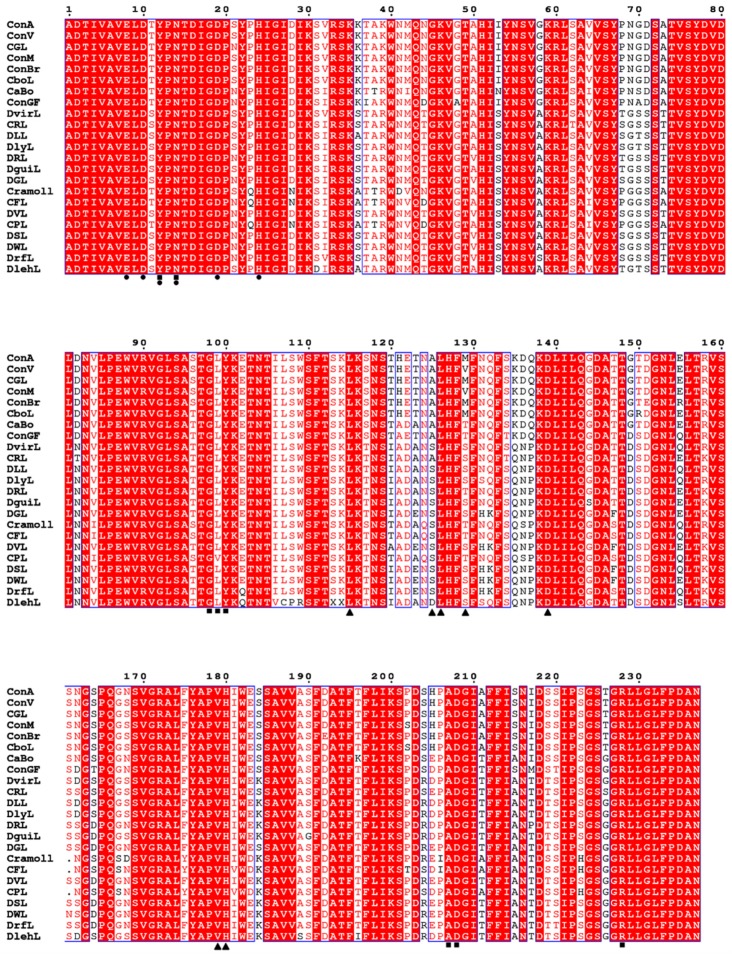
Multiple alignment of the amino acid sequence of Diocleinae lectins including sugar-binding residues (square), metal-binding residues (circles), and hydrophobic site residues (triangle). These lectins have high sequence homology (>80%, relative to ConA).

ConA-like lectins are composed of 237 amino acid residues with few exceptions, such as CPL, Cramoll and CFL that have 236 residues showing a deletion at position 161 in relation to the other lectins (Figure 1). These lectins attain this form from a post-translational processing of their glycoprotein precursors, which are 290 amino acids (aa), known as circular permutation. The prepro-lectin (signal + γ-chain + central peptide + β-chain + Cterm) is synthesized in seed cotyledons and accumulated in reserve parenchymal cells. This is addressed to the endoplasmic reticulum where the signal peptide is removed, and the central peptide is *N*-glycosylated, forming the pro-lectin. In Golgi complex, structural changes occur in its glycan, and then it is transported to the vacuoles. In the vacuoles, the pro-lectin is processed by asparaginyl endopeptidases and carboxypeptidases which cleave the central glycopeptide containing 15 aa and the Cterm region, resulting in the loss of 9 aa. The γ- and β-chains are relegated in the reverse order forming an α-chain (β + γ), which consists of the lectin mature chain of ~237 aa with an average molecular mass of 25 kDa. This processing was initially described for ConA as circular permutation [8,105,106,107,108,109].

**Figure 2:**
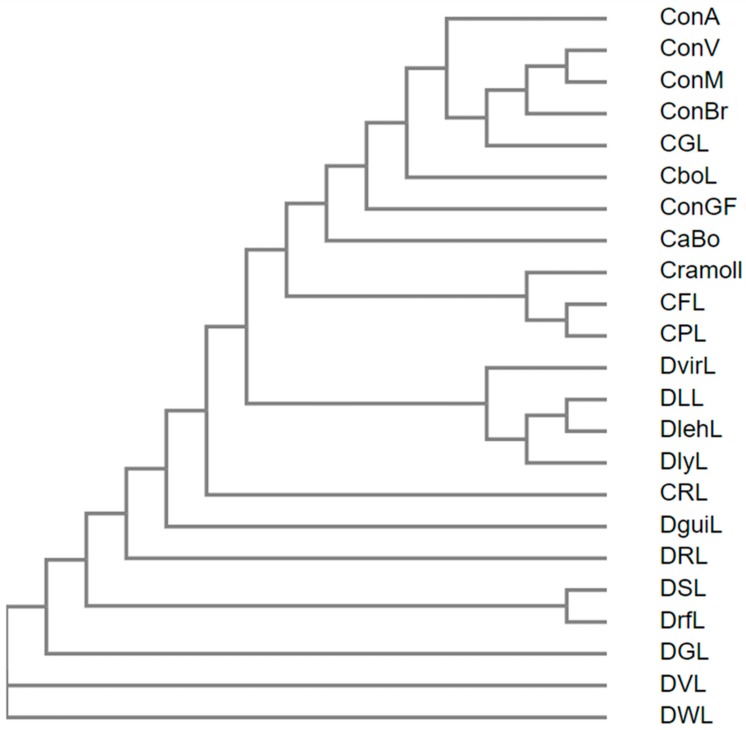
Phylogenetic tree shows the evolutionary scale between Diocleinae lectins.

Approximately 67% of the residues (159 aa) are conserved in all lectins, those being 35.44% in the β-chain (84 aa) and 31.64% in the γ-chain (75 aa). Some differences are very punctual, but some segments, such as 68–71, 117–125 and 135–137, have sequence peculiarities between lectins of the *Canavalia* genus and other Diocleinae lectins. In addition, region 117–125 connects the β and γ chains. DVL and DWL are the most primitive lectins of the Diocleinae subtribe, whereas ConV, ConM, CFL, CPL, DLL, DlehL, DSL and DrfL are evolutionarily more recent. The phylogenetic profile of Diocleinae lectins is shown in Figure 2.

MBS and CRD residues are conserved in most lectins, except CPL, which has a substitution of leucine for valine at position 99. Most residues of the hydrophobic site are conserved with the exception of residues at positions 125 and 129 that vary between proteins. More details about them are discussed in the next section.

### 5.2. Three-Dimensional Structure

In 1972, Hardman and Ainsworth [110] determined the crystallographic structure of ConA at a resolution of 2.4 Å. This was the first Diocleinae lectin to have its three-dimensional structure characterized. Since then, several other ConA-like lectins have had their three-dimensional structures determined, and, today, they total more than 100 deposits in the Protein Data Bank. With the exception of DlehL, all lectins cited in the previous section have had their three-dimensional structure determined and have been deposited in databases (Table 4).

**Figure 3:**
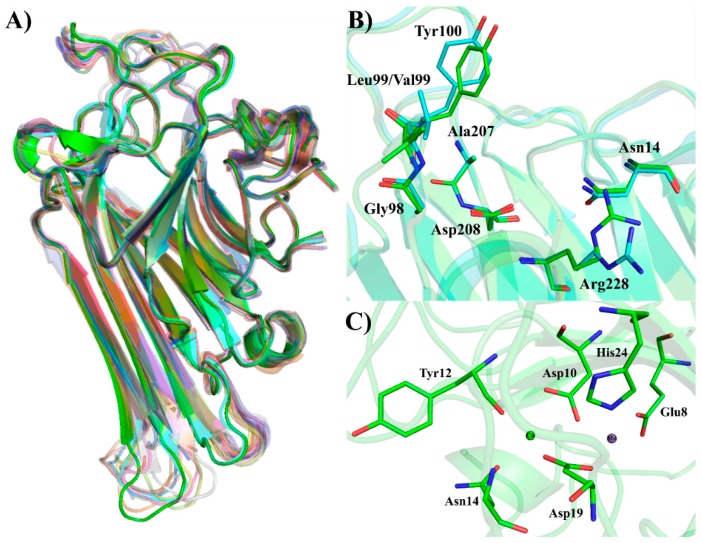
(**A**) Superposition of Diocleinae lectins monomers. The structures of ConA (green), ConV (cyan), CGL (pink), ConM (yellow), ConBr (orange), CboL (gray), CaBo (purple), ConGF (red), DvirL (blue), CRL (dark green), DLL (palecyan), DlyL (dark red), DRL (violet), DguiL (brown), DGL (marine blue), Cramoll (gold yellow), CFL (salmon), DVL (lightblue), CPL (lightgreen), DSL (lightorange), DWL (lightpink) and DrfL (darkgray) are represented in cartoon; (**B**) carbohydrate recognition domain of ConA (green) and CPL (cyan); and (**C**) representations of ConA metal-binding site with Ca^2+^ and Mn^2+^.

The monomer of these proteins is characterized by the presence of β-sandwich folding, also known as a jellyroll motif. This motif is observed in many leguminous lectins, but it could also be seen in viral capsid proteins. This folding consists of two antiparallel β-sheets connected by loops. One contains six strands and is partially extended, and the other is curved and has seven strands. The folding is stabilized by noncovalent interactions between the β-sheets and two hydrophobic cores [111,112,113]. Figure 3A shows the overlap of ConA-like lectin monomers made in Pymol software. The Cα RMSD values are recorded in Table 5 and were obtained using ConA as the reference structure. Both the overlap analysis and the low Cα RMSD values indicate that the jellyroll motif is conserved in these proteins and small variations can be usually found in loop regions.

Three types of binding sites were identified in these lectins, the carbohydrate recognition domain (CRD), the metal-binding site (MBS) and the hydrophobic site (HS). The CRD is composed of four discontinuous segments present on the curved β-sheet of the jellyroll, including 12–16, 98–100, 207–208 and 227–229. Amino acid residues that directly participate in the interaction with carbohydrates are Tyr12, Asn14, Gly98, Leu99 (Val99, in the case of CPL), Tyr100, Ala207, Asp208 and Arg228. MBS is also present in the curved B-sheet and has two coordinated divalent cations, calcium and manganese. Six amino acid residues and four water molecules participate in octahedral type coordination. Calcium is coordinated by residues Asp10, Tyr12, Asn14 and Asp19, while manganese is coordinated by residues Glu8, Asp10, Asp19 and His24, with two molecules coordinating each ion. MBS stabilizes a cis-peptide in the Ala207–Asp208 residues, which is crucial for CRD formation. Through a water bridge, the calcium ion can interact with the main chain atoms of that cis-peptide, which is at the base of the CRD and is responsible for stabilizing the carbohydrates within the site cavity [113]. The importance of this cis-peptide was demonstrated through structural studies by crystallography and molecular modeling, as well as molecular docking and dynamics involving CaBo, DlyL and DrfL [44,52,114]. Figure 3B,C demonstrates both sites for four Diocleinae lectins.

The HS was first described for CGL by Delatorre and colleagues (2007) [115] who co-purified the lectin with alpha-aminobutyric (Abu), a non-protein amino acid. After diffraction of CGL crystals, it was possible to observe Abu electron density in a hydrophobic pocket formed by residues Leu115, Leu126 and Val179. Abu also forms hydrogen interactions with Ala125 and His180. HS is present in the interdimeric region; thus, Abu could also form hydrogen interactions with residues of the adjacent monomer, involving residues Met129 and Asp139. Similar results were seen with ConBr in the study of Bezerra et al. (2011) [49]. Since most HS residues are conserved in other Diocleinae lectins, they are also likely carriers of hydrophobic molecules.

### 5.3. Quaternary Structure

**Figure 4:**
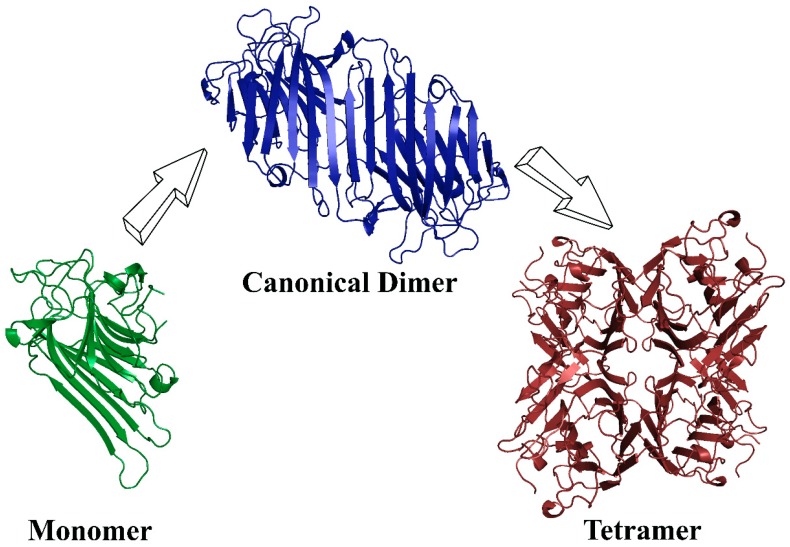
Scheme of ConA-like lectins oligomerization.

The monomers of Diocleinae lectins can interact with each other through residues present in the partially extended β-sheet, forming dimers and tetramers. The dimers of these proteins are canonical; that is, the monomers associate adjacently, forming a long β-sheet of 12 strands (six per monomer). The tetramer is formed by the association of two canonical dimers; in other words, it is a dimer of dimers, generating a tetramer with an interface of X2 [116,117]. A scheme of the ConA-like lectins oligomerization is shown in Figure 4.

The oligomerization of Diocleinae lectins can be affected by the pH of the medium, which, in turn, determines whether the lectin will be in its dimeric or tetrameric form, a property termed pH-dependent oligomerization. All lectins of the *Canavalia* genus have pH-dependent oligomerization, while, for other ConA-like lectins, this depends on particular structural features [118]. The first report on the divergence of pH-dependent oligomerization among ConA-like lectins was from Calvete et al. (1999) [101] who analyzed the sedimentation by ultracentrifugation of these proteins at different pH values and observed heterogeneous results with some lectins, such as DGL and DVL, that did not present a sedimentation pattern similar to that of ConA [119,120], indicating pH-independent oligomerization. The first study to explain this property at the structural level was published by Wah et al. (2001) [121], who studied DguiL, a lectin with pH-dependent oligomerization. Based on the crystallographic structure of DguiL, it has been observed that the Asn131 residue of a monomer does not interact with loop 114–125 of the opposite monomer located on the central cavity when the lectin is in its tetrameric form. A dramatic reduction in interdimeric contacts occurs in the absence of this interaction in DguiL, but not in DGL, a lectin with pH-independent oligomerization. In this case, a histidine in region 131 establishes interactions in the central cavity toward increasing interdimeric contacts. A primary structure analysis demonstrated that lectins bearing His131 are pH-independent and those having other residues at this position are pH-dependent. This report established a new structural basis for this phenomenon and replaced the work of Senear and Teller (1981) [122], who used sedimentation analysis by ultracentrifugation in ConA. They concluded that the oligomerization of ConA was dependent on the protonation of the His51 and His121 residues.

Further studies of Gallego del Sol et al. (2007) [123] with CFL and both Nagano et al. (2008) [12] and Zamora-Caballero et al. (2015) [124] with native and recombinant mutants forms of DguiL and DGL demonstrated the importance of interactions between residues occurring in the central cavity and in peripheral regions for the formation of stable tetramers by combining techniques involving X-ray crystallography, site-directed mutations and ultracentrifugation sedimentation. These studies increased the structural bases of oligomerization, as well as correlated the data obtained by Senear and Teller (1981) [122] and Wah et al. (2001) [121]. They concluded that the His51 residue contributes to the stabilization of the 114–125 loop in the central cavity, providing a favorable spatial orientation for the interaction with His131, which increases the interdimeric contacts that stabilize the tetramer. Substitutions on one of these two residues did not affect the pH-independent effect of DGL oligomerization, but the mutation in both effectively abolished tetrameric formation. In peripheral regions, residues Arg60 and Asp78 are also responsible for the stabilization of the quaternary form. Mutations in this region, together with His51, were able to abolish the tetrameric formation of DGL. This demonstrates a greater complexity of the molecular bases that influence oligomerization. In addition, pH defines the protonation states of all cited residues which also include the electrostatic factor that influences interdimeric contacts.

## 6. Physicochemical Properties

Determination of physicochemical properties of lectins usually includes their thermostability, stability at different pH and dependence of divalent cations, all obtained through hemagglutination tests with lectin solution at different conditions. ConA-like lectins have fairly similar physicochemical properties and can be considered stable since they support a wide range of pH and temperatures without loss of their activities. Most of these lectins maintain their activity in the range of pH 5–8 with maximum activity in the range of pH 7–8. These proteins normally maintain their maximum activity up to 60 °C, but above that temperature, activity is lost by denaturation until approximately 70–80 °C when total loss of hemagglutinating activity occurs [17,18,19,20,51,125].

Dependence on divalent cations is routinely tested owing to the metalloprotein nature of legume lectins. These proteins usually have Ca^2+^ and Mn^2+^ ions in a metal-binding site (MBS) located in the vicinity of the carbohydrate-recognition domain (CRD). These ions are important for CRD stabilization, thus allowing lectin binding to carbohydrates [36,113]. More details on the MBS can be found in Section 5.2. High losses in activity after treatment with EDTA can be observed for most characterized ConA-like lectins; thus, divalent cations appear to be a requirement for their activity [18,20,51]. CoxyL, ConV and ConC are exceptions since treatment of these lectins with the chelating agent does not affect their hemagglutinating activity [17,19,125].

When applied in polyacrylamide gel electrophoresis in the presence of SDS, ConA-like lectins presented three bands corresponding to α, β, and γ polypeptide chains with relative molecular masses of approximately 25, 14 and 12 kDa, respectively. These chains result from the circular permutation processing suffered by these lectins, as previously described in Section 5.1. None of the ConA-like lectins to date is glycoprotein in nature, as revealed by periodic-acid Schiff staining and mass spectrometry of several lectins.

## 7. Structure/Biological Activities Relationships

CRD residues are conserved in most ConA-like lectins, but they have different patterns and intensity of interaction with ligands. These differences may be related to the different effects promoted by these lectins in biological activity assays, even those having high homology of primary structure [8]. One way of evaluating this difference is through the use of volume analysis of CRD via crystallographic structures, which demonstrated that CRD volume and cavity entrance area are not uniform in these lectins [49,53,54,56,99]. This was best evidenced in molecular dynamics studies with DrfL and DSL, in which the CRD volume was monitored during the simulation [52].

In addition, molecular docking simulations with the CaBo, DrfL, DlyL and DLL lectins have suggested the ability of these lectins to interact with high-mannose, complex and hybrid *N*-glycans [52,59,61,126]. Many of these tested carbohydrates are anchored covalently to an asparagine residue in glycoproteins and glycoconjugates present on the cell surface, and structural modifications in their compositions are observed in tumor cells [127,128,129,130,131]. Modified *N*-glycans can affect the survival and progression of cancer cells, as well as accelerate metastatic processes [132,133,134]. As another example, high mannose *N*-glycans are present in gp120 glycosylation in the HIV envelope, and they are essential for HIV attachment to cell-surface receptors, such as CD4 (cluster of differentiation 4) [135,136]. Furthermore, oligomannosides can be found on the surface of endothelial cells and are involved in many cell–cell recognition processes, such as leukocyte recruitment. They can also be anchored to glycoproteins involved in some processes of the immune system [137,138,139]. Experimental interaction data, as well as docking results, suggest that Diocleinae lectins can interact with these glycans, which may explain many of their biological effects [14,52,59,61,126].

The pH-dependent oligomerization effect can also be considered one of the factors that can influence the biological effects elicited by these lectins since dimer-tetramer equilibrium has an impact on CRD conformation and that can affect the interaction of these proteins with carbohydrates/glycosylated receptors [140,141]. Experimental results from the study of Mandal and Brewer (1993) [140] demonstrated that the dimeric and tetrameric forms may have differentiated affinities for glycans. The authors observed that Man7, Man8 and Man9 oligomannose-type glycopeptides have reduced affinities for dimeric form in comparison with the tetrameric form. On the other hand, both dimeric and tetrameric ConA presented the same affinity for mannose, dimmanosides and Man3 glycopeptide. Altogether, this strongly indicates that that oligomerization state appears to confer to these lectins a variability in specificity for oligosaccharides or glycoconjugates which may be key molecules for a biological activity.

## 8. Conclusions

Lectins comprise a valuable group of proteins with applications in several areas. Inside the lectins, those from the Diocleinae subtribe, usually called ConA-like lectins, are a group of proteins with high similarities at amino acid sequence level and three-dimensional structure level. These proteins have long been objects of study, and many of them were extensively studied. ConA-like lectins present several biological activities that are described in this review. Despite the high similarity among ConA-like lectins, their affinity for carbohydrates is variable, and their biological activities also differ from one lectin to another. We found that such variability typically results from small sequence differences that affect the CRD region and the pH-dependent oligomerization exhibited by some of these proteins. However, this same characteristic enables the use of these proteins in the study of structure/biological activities relationships.

## Figures and Tables

**Table 1:** Comparison of pro-inflammatory activities of Diocleinae lectins.

Lectin	Initial	Edema Peak	Edema (mL)	Duration	Indomethacin	L-NAME ^a^	Carbohydrate ^b^
ConA	1 h	1 h	0.29	24 h	NT ^c^	NT	Partially
ConBr	30 min	30 min	0.92	48 h	Partially	Partially	Partially
CGL	30 min	30 min	0.68	32 h	Partially	Partially	Partially
ConM	30 min	30 min	0.56	5 h	Partially	No	Blocked
ConGF	30 min	30 min	0.41	24 h	NT	NT	NT
CoxyL	30 min	2 h	1.10	5 h	NT	NT	NT
DWL	30 min	5 h	0.80	24 h	Partially	Partially	No
DlyL	30 min	1 h	1.06	24 h	Partially	Partially	No
CRL1	3 h	4h	0.47	24 h	NT	NT	Partially

^a^ N(ω)-nitro-L-arginine methyl ester; ^b^ Lectin-specific carbohydrate that was used to inhibit its activity; ^c^ Not tested.

**Table 2:** Comparison of vasorelaxation effects of Diocleinae lectins.

Lectin	Endothelium-Dependent	Relaxation	Indomethacin	L-NAME ^a^	Carbohydrate ^b^
ConBr	Yes	74%	Partially	Partially	Partially
CGL	Yes	108%	Partially	Blocked	Partially
ConGF	Yes	25%	NT ^c^	Blocked	NT
ConM	Yes	110%	NT	Blocked	NT
ConA	Yes	85%	NT	NT	NT
ConV	Yes	77%	NT	NT	Partially
CRL1	Yes	96%	No	Blocked	NT
DLL	Yes	81%	NT	Partially	Partially
DrfL	Yes	32%	NT	Blocked	Partially
DSL	Yes	36%	NT	Blocked	Blocked
DRL	Yes	96%	Partially	Blocked	Partially
DVL	Yes	43%	No	Partially	Partially
DvirL	Yes	70%	NT	Blocked	NT

^a^ N(ω)-nitro-L-arginine methyl ester; ^b^ Lectin-specific carbohydrate that was used to inhibit its activity; ^c^ Not tested.

**Table 3:** Cytotoxic concentration of Diocleinae lectins for 50% of glioma cell viability (CC_50_).

Lectin	CC 50 (µg/mL)	Confidence Interval
ConA	56.02	38.01–80.03
CaBo	230.50	59.84–88.8
ConV	58.8	52.45–140.3
DVL	58.84	51.14–67.70
DLL	70.51	58.32–85.25

**Table 4:** List of deposits IDs of Diocleinae lectins structures in the PDB and Uniprot database.

Lectin	PDB ID	Uniprot ID
ConA	1JBC	P02866
ConV	5F5Q	C0HJY1
CGL	1WUV	P14894
ConM	2CWM	P81364
ConBr	1AZD	P55915
CboL	4K20	A0A023GPI8
CaBo	5U3E	P58906
ConGF	4L8Q	A0A067XG71
DvirL	3RRD	P58907
CRL	3A0K	P86184
DLL	5UUY	-
DlyL	6CJ9	C0HK27
DRL	2ZBJ	P58908
DguiL	1H9W	P81637
DGL	1DGL	P08902
Cramoll	1MVQ	P83721
CFL	2D3P	P81517
DVL	2GDF	I1SB09
CPL	3U4X	J9PBR3
DSL	4NOT	B3EWJ2
DWL	3SH3	P86624
DrfL	5TG3	C0HK81
DlehL	-	-

**Table 5:** Carbon alpha RMSD values of Diocleinae lectins using ConA structure (PDB ID: 1JBC) as reference.

Lectin	Cα RMSD
ConV	0.260
CGL	0.346
ConM	0.304
ConBr	0.310
CboL	0.299
CaBo	0.335
ConGF	0.467
DvirL	0.322
CRL	0.467
DLL	0.290
DlyL	0.283
DRL	0.287
DguiL	0.276
DGL	0.368
Cramoll	0.331
CFL	0.330
DVL	0.369
CPL	0.270
DSL	0.280
DWL	0.351
DrfL	0.312

## Abbreviations

ConA	Canavalia ensiformis lectin
CRD	Carbohydrate-recognition domain
Man	Mannose
SBA	Soybean agglutinin
ConGF	*Canavalia grandiflora* lectin
PEG	Polyethylene glycol
CGL	*Canavalia gladiata* lectin
ConM	*Canavalia maritima* lectin
ConBr	*Canavalia brasiliensis* lectin
ConV	*Canavalia virosa* lectin
CoxyL	*Canavalia oxyphylla* lectin
CvilL	*Canavalia villosa* lectin
DRL	*Dioclea rostrata* lectin
DWL	*Dioclea wilsonii* lectin
DvirL	*Dioclea virgata* lectin
DlyL	*Dioclea lasiophylla* lectin
DrfL	*Dioclea reflexa* lectin
CRL I	*Cymbosema roseum* lectin
DguiL	*Dioclea guianensis* lectin
DVL	*Dioclea violacea* lectin
CFL	*Cratylia floribunda* lectin
L-NAME	N(ω)-nitro-L-arginine methyl ester
DSL	*Dioclea sclerocarpa* lectin
eNOS	Endothelial nitric oxide synthase
DLL	*Dioclea lasiocarpa* lectin
IFN-γ	Interferon-γ
IL	Interleukin
TNF-α	Tumor Necrosis Factor-α
NMDA	N-methyl-D-aspartate
DlehL	*Dioclea lehmani* lectin
SDS	Sodium dodecyl sulfate
HS	Hydophobic site
MBS	Metal-binding site
CaBo	*Canavalia bonariensis* lectin
ConBol	*Canavalia boliviana* lectin
Cramoll	*Cratylia mollis* lectin
CPL	*Camptosema pedicallatum* lectin
CNS	Central Nervous System
NO	Nitric Oxide
